# Association between physical activity levels and lower urinary tract symptoms: a cohort study in 20,732 Brazilian men

**DOI:** 10.31744/einstein_journal/2024AO1204

**Published:** 2024-11-26

**Authors:** Rafael Mathias Pitta, Oskar Kaufmann, Raphael Mendes Ritti-Dias, Luana de Lima Queiroga, Nelson Wolosker

**Affiliations:** 1 Hospital Israelita Albert Einstein Faculdade Israelita de Ciências de Saúde Albert Einstein São Paulo SP Brazil Programa de Pós-Graduação em Ciências da Saúde, Faculdade Israelita de Ciências de Saúde Albert Einstein, Hospital Israelita Albert Einstein, São Paulo, SP, Brazil.; 2 Universidade Nove Julho São Paulo SP Brazil Programa de Pós-Graduação em Ciências da Reabilitação, Universidade Nove Julho, São Paulo, SP, Brazil.; 3 Universidade de São Paulo Faculdade de Medicina São Paulo SP Brazil Faculdade de Medicina, Universidade de São Paulo, São Paulo, SP, Brazil.; 4 Hospital Israelita Albert Einstein São Paulo SP Brazil Hospital Israelita Albert Einstein, São Paulo, SP, Brazil.

**Keywords:** Lower urinary tract symptoms, Exercise, Lifestyle, Erectile dysfunction

## Abstract

**Objective:**

This study aimed to evaluate the association between physical activity levels and lower urinary tract symptoms in 20,732 Brazilian men.

**Methods:**

This cohort study included 20,732 men of ≥40 years of age, who participated in health screening between January 2008 and December 2018. Standardized health data (anthropometric, laboratory, clinical, and lifestyle data) were collected. A logistic regression model was used to examine associations based on the occurrence of lower urinary tract symptoms.

**Results:**

Depressive symptoms (p<0.001), age (p<0.001), erectile dysfunction (p<0.001), and total prostate-specific antigens (p<0.001) were associated with higher odds of lower urinary tract symptoms in men, after adjusting the other factors. However, physical activity levels were associated with lower odds of developing lower urinary tract symptoms (low active, p<0.001; active, p=0.002; and high active, p=0.005).

**Conclusion:**

All physical activity levels were associated with lower odds of lower urinary tract symptoms in 20,732 Brazilian men.

## In Brief

Pitta et al. demonstrated that all physical activity levels were associated with lower odds of lower urinary tract symptoms in 20,732 Brazilian men and should be strongly encouraged by public authorities and private clinicians during the treatment and prevention of this condition.

## Highlights

■ Any physical activity level decreased the odds of lower urinary tract symptoms.
■ Aging and the occurrence of depression, erectile dysfunction, and higher total prostate-specific antigens were associated with higher odds of lower urinary tract symptoms.

## Graphical abstract

**Figure:**
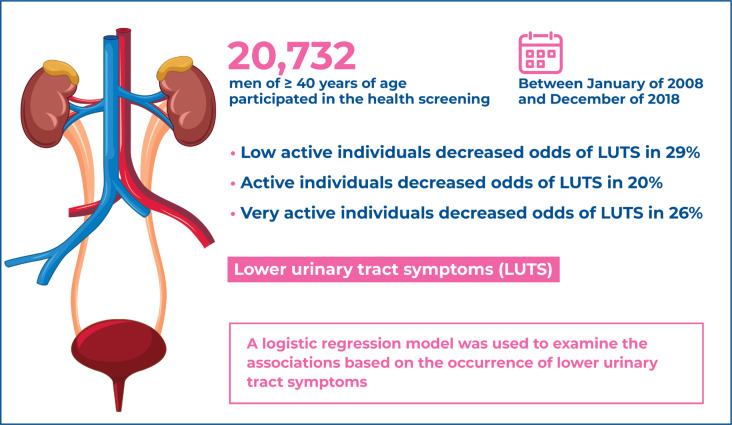

## INTRODUCTION

Lower urinary tract symptoms (LUTS) is a common condition that results from conditions and diseases affecting the bladder and urethra, including urinary incontinence (stress, urge, and mixed urinary incontinence), storage/overactive bladder symptoms (urgency, frequency, and nocturia, with or without incontinence), voiding (urinary retention, hesitancy, straining to void, and slow or interrupted stream), and postmicturition (postmicturition dribble).^(1,2)^ Lower urinary tract symptoms affects approximately 20% of men of >40 years of age,^(1)^ 50-70% of men >50 years,^(2)^ and 80% of men >70 years, and is commonly associated with erectile dysfunction and cardiovascular diseases, leading to a lower quality of life,^(2)^ which makes it a significant public health problem and economic burden on society.

Despite extensive research in this area, based on our knowledge, the extended underlying pathophysiology leading to LUTS is unclear. Kim et al.^(3)^ reported that lower physical activity levels were associated with overall and moderate LUTS. Additionally, voiding symptoms were closely associated with lower levels of physical activity. However, in previous studies,^(4–6)^ physical activity status was associated with an improvement in LUTS among middle-aged individuals. In addition, the development of preventive measures to reduce the burden of LUTS by identifying the risk factors associated with these symptoms, especially those that are potentially modifiable, such as physical activity, is gaining popularity.^(7)^ Various studies have shown that different levels of physical activity are associated with a reduced risk of developing LUTS^(7,8)^ and sedentary behavior was associated with developing LUTS in a large cohort of men without LUTS;^(9)^ however, the results of physical activity were unclear. Although statistically significant, the association between physical activity and LUTS may be an incidental finding in this population sample. After adjusting the model, low active and active individuals were associated with lower odds of LUTS than sedentary individuals. Additionally, the study comprised middle-aged men and only 162 men >65 years of age, making it difficult to understand the association between physical activity and LUTS in the older population. Age, number of comorbidities, medication use, higher body mass index (BMI), lower socioeconomic status, alcohol consumption, smoking status, ethnicity, and cultural factors influence LUTS^(1,2,7–9)^ and should be considered in its multifactorial pathology.

Although the effectiveness of physical activity has been confirmed,^(7,8)^ other variables may have influenced its effects. Initial clinical conditions, such as LUTS and non-LUTS, affect the effects of physical activity on the incidence and reduction of LUTS. Only one study^(10)^ has performed multivariable analyses (adjusted for confounding factors) that considered clinical and modifiable lifestyle factors in a large cohort of men. However, the association between physical activity and LUTS after adjusting for factors is not clear in relation to different physical activity levels, volumes, and intensities, similar to the lack of clarity in the role of other important clinical factors in this context.

To the best of our knowledge, no study has compared individuals with and without LUTS based on their physical activity levels and clinical outcomes (hypertension, diabetes, non-alcoholic fatty liver disease, smoking, alcohol consumption, and anxiety). Lower urinary tract symptoms is highly associated with the burden of chronic diseases in developing countries^(1,2)^ and different clinical conditions can affect physical activity levels and clinical outcomes.

## OBJECTIVE

This cohort study aimed to evaluate the association between lower urinary tract symptoms and physical activity levels in a large, representative sample of Brazilian adults and older adults.

## METHODS

All participants underwent a standardized health examination that included an assessment of the prevalence and severity of LUTS and physical activity levels, reinforcing the reliability and validity of the findings.

This cohort study's primary outcome was to identify an association between physical activity levels and LUTS in 20,732 Brazilian men of ≥40 years of age based on the data collected from a health assessment in the Preventive Medicine Center at *Hospital Israelita Albert Einstein* (São Paulo, Brazil) between 2008 and 2018. The study was approved by the hospital's ethics committee (CAAE: 94867018.6.0000.0071; # 2.844.247), and the requirement for informed consent was waived.

The database initially included data from 44,395 Brazilian men of ≥40 years of age who underwent health checkups. Duplicate data, including data from individuals who had undergone more than two health screenings, were excluded. Participants with missing physical activity or LUTS data were also excluded. Finally, the data on physical activity and LUTS from 20,732 men of ≥40 years of age were analyzed.

### Data collection

#### Clinical data

This investigation consisted of questionnaires, anthropometric measurements, and laboratory tests conducted by trained interviewers (doctors, nutritionists, nurses, physiotherapists, physical educators, and psychologists).

Weight was recorded using the InBody 230 equipment (Ottoboni^®^), and height was measured using a stadiometer. Subsequently, BMI was calculated as follows: BMI (kg/m²)=weight/(height × height)².

Systolic and diastolic blood pressure (mg/dL) were recorded based on triplicate measurements according to the American Heart Association^(11)^ after the patients had rested for at least 10 minutes. The measurements were conducted on both arms using the auscultatory method with an aneroid sphygmomanometer and Korotkoff sounds of phases I and V. If the necessary data were not available for medical assessment, we considered the presence of hypertension and diabetes based on self-reported information from the patients, including the chronic use of antihypertensive medications, self-reported *diabetes mellitus*, or self-reported chronic use of anti-diabetic medications.

Metabolic syndrome was defined according to the World Health Organization recommendations.^(12)^ Comorbidities (systemic arterial hypertension, *diabetes mellitus*, dyslipidemia, tobacco use, non-alcoholic fatty liver steatosis, and continuously used medications) were assessed using medical records. Laboratory data (glycosylated hemoglobin percentage [HbA1C, %], standard lipid panel [mg/dL], creatinine [mg/dL], thyroid-stimulating hormone [TSH, mU/L], and total prostate-specific antigen [PSA, ng/mL]) were collected after an overnight fast and standardized criteria for quality control were established by the Brazilian Health Ministry. Ultrasensitive C-reactive protein (CRP, mg/dL) levels were measured using a turbidimetric method on a nephelometry system (Dade-Boehring, EUA).

Standardized questionnaires were used to analyze lifestyle habits. Alcohol consumption was evaluated using the Alcohol Use Disorders Identification test,^(13)^ the presence and severity of depressive symptoms using the Beck Depression Inventory-II,^(14)^ perceived stress using the Perceived Stress Scale-10,^(15)^ and erectile dysfunction using the International Index of Erectile Function-5.^(16)^

### Physical activity

Physical activity levels were assessed using the International Physical Activity Questionnaire (IPAQ).^(17)^ IPAQ provides information on volume (time spent), intensity (light, moderate, and vigorous), and sedentary activity in a usual week and classifies participants into four levels: highly active, active, low active, and physically inactive. Participants who engage in at least 30 minutes of vigorous physical activity for at least 5 days per week or those who engage in at least 20 minutes of vigorous physical activity for at least 3 days per week and/or walking for at least 30 minutes for at least 5 days per week were classified as highly active. Individuals who practice at least 20 minutes of vigorous physical activity at least 3 days a week or those who practice any physical activity for at least 150 minutes a week spread out over at least 5 days were considered active. Individuals who reported engaging in physical activity but did not meet the above criteria were classified as low active. Those who reported no physical activity were considered physically inactive.

### Lower urinary tract symptoms

Doctors assessed the presence and severity of LUTS using the International Prostate Symptom Score (IPSS).^(18)^ The questionnaire consists of eight questions on two outcomes (seven on benign prostatic hyperplasia and one on quality of life) and is scored on a six-point ordinal scale (0-5), where lower values represent poorer function. The LUTS scoring criteria are: 0-7, absent/mild; 8-19, moderate; and 20-35, severe. In this study, the presence of LUTS was defined based on scores ≥8, including severe and moderate categories. The absence of LUTS was defined based on a score <8.

Initially, we compared the clinical and lifestyle variables related to LUTS. Subsequently, we compared the demographic, comorbidity, and lifestyle variables associated with LUTS. Finally, we performed stepwise backward multiple logistic regression analysis and analyzed the variables associated with LUTS.

### Statistical analysis

Shapiro-Wilk test was used to assess data normality. Descriptive statistics are expressed as mean and standard deviation for continuous variables and frequency and percentage for categorical variables. We used chi-squared test to compare categorical variables and analysis of variance and Bonferroni post hoc test for continuous variables. A logistic regression model was used based on the occurrence of LUTS. Laboratory data, age, BMI, hypertension, diabetes, tobacco use, dyslipidemia, alcohol consumption, perceived stress, depressive symptoms, erectile dysfunction, and physical activity were used as covariates in logistic regression models. Adjusted odds ratios (aORs) and 95% confidence intervals (95% CIs) were computed for logistic model results. A p<0.05 was considered statistically significant. Statistical analyses were performed using SPSS for Windows (version 24.0; IBM Corp., Armonk, NY, USA).

## RESULTS

We analyzed the data of 20,732 men 40-91 years of age. A total of 19,017 (91.7%) patients had absent or mild LUTS, 1,521 (7.3%) had moderate LUTS, and 194 (0.9%) had severe LUTS. Furthermore, 20.3%, 20%, 39.4%, and 11.4% of patients were categorized as sedentary, low active, active, and high active, respectively.

Table 1 presents a comparison of the demographic, anthropometric, and laboratory data according to LUTS. Individuals with moderate and severe LUTS were older than those with absent/mild LUTS (55.22±8.86, 57.41±9.12, and 49.58±7.24 years, respectively; all p<0.001) and BMI was significantly higher in men with severe LUTS than absent/mild LUTS (28.47±4.12 *versus* 27.74±3.99kg/m², p<0.001). Total cholesterol was significantly higher in men with absent/mild LUTS than those with moderate and severe LUTS (195.38±38.85, 186.34±38.35, and 188.01±40.00mg/dL, respectively; all p<0.001). Low-density lipids were significantly higher in men with absent/mild LUTS than moderate LUTS (121.35±34.18 *versus* 114.57±34.01mg/dL, p<0.001). Finally, patients with moderate and severe LUTS had higher HbA1C than absent/mild LUTS (5.73±0.97%, 5.89±1.11%, and 5.59±0.72%, respectively; all p<0.001). Total PSAs were significantly higher in men with moderate and severe LUTS than absent/mild LUTS (1.74±4.10, 2.04±2.43, and 1.05±1.76ng/mL, respectively; all p<0.001) and ultrasensitive CRP was significantly higher in men with moderate LUTS than absent/mild LUTS (3.36±12.17 *versus* 2.57±5.28mg/dL, p<0.001).

**Table 1:** Demographic and laboratory test results in relation to lower urinary tract symptoms

Variable	IPSS	Mean	SD	Median	Minimum	Maximum	n	p value*†
Age	Absent/mild	49.58	7.24	48	40	91	19,017	<0.001*†
	Moderate	55.22	8.86	55	40	90	1521	
	Severe	57.41	9.12	57.5	41	88	194	
	Total	50.06	7.57	49	40	91	20,732	
BMI (kg/m^2^)	Absent/mild	27.74	3.99	27.16	13.11	55.50	19,014	0.003†
	Moderate	27.98	4.09	27.41	17.40	50.06	1,521	
	Severe	28.47	4.12	28.09	20.45	53.30	197	
	Total	27.76	4.00	27.17	13.11	55.50	20,732	
TC (mg/dL)	Absent/mild	195.38	38.85	193	55	500	19,014	<0.001*†
	Moderate	186.34	38.35	186	78	406	1,522	
	Severe	188.01	40.00	188	91	303	196	
	Total	194.65	38.90	193	55	500	20,732	
HDL (mg/dL)	Absent/mild	45.52	11.18	44	9	148	19,014	0.139
	Moderate	45.28	10.90	44	9	93	1,522	
	Severe	44.05	10.02	43	25	80	196	
	Total	45.49	11.15	44	9	148	20,732	
TG (mg/dL)	Absent/mild	150.37	112.60	126	18	5,008	19,007	0.090
	Moderate	143.95	88.75	121	28	1,550	1,530	
	Severe	151.94	74.85	131.5	51	474	195	
	Total	149.92	110.74	126	18	5,008	20,732	
LDL (mg/dL)	Absent/mild	121.35	34.18	120	10	409	19,006	<0.001*
	Moderate	114.57	34.01	115	27	339	1,530	
	Severe	116.38	35.10	116	33	217	196	
	Total	120.81	34.22	120	10	409	20,732	
HbA1c (%)	Absent/mild	5.59	0.72	5.50	3.70	13,60	17,440	<0.001*†
	Moderate	5.73	0.97	5.50	4	13,90	2,344	
	Severe	5.89	1.11	5.60	4.5	11.70	948	
	Total	5.61	0.75	5.50	3.70	13.90	20,732	
Total PSA (ng/dL)	Absent/mild	1.05	1.76	0.77	0	168	18,944	<0.001*†
	Moderate	1.74	4.10	1.03	0	134	1,586	
	Severe	2.04	2.43	1.18	0	18.85	212	
	Total	1.11	2.04	0.79	0	168	20,732	
TSH (mU/L)	Absent/mild	2.45	2.76	2.07	0	167	18,839	0.975
	Moderate	2.46	1.55	2.17	0	17.20	1,679	
	Severe	2.45	1.35	2.13	0.10	6.83	214	
	Total	2.45	2.68	2.08	0	167	20,732	
Ultrasensitive c-reactive protein (mg/dL)	Absent/mild	2.57	5.28	1.3	0	148.40	19,536	0.020*
	Moderate	3.36	12.17	1.4	0	192.40	1,117	
	Severe	2.90	2.95	1.9	0	11.70	79	
	Total	2.60	5.69	1.3	0	192.40	20,732	
Creatinine (mg/dL)	Absent/mild	0.94	0.18	0.92	0.32	12.45	18,927	0.532
	Moderate	0.94	0.20	0.91	0.49	3.30	1,570	
	Severe	0.93	0.15	0.9	0.6	1.54	235	
	Total	0.94	0.18	0.92	0.32	12.45	20,732	

P-values are based on analysis of variance and Bonferroni post-hoc tests;

* absent/mild vs. moderate LUTS;

† absent/mild versus severe LUTS.

LUTS: lower urinary tract symptoms; IPSS: International prostate symptom score; SD: standard deviation; n: sample size; BMI: body mass index; TC: total cholesterol; HDL: high-density lipid; TG: triglyceride; LDL: low-density lipids; HbA1c: glycosylated hemoglobin; PSA: prostate-specific antigen; TSH: thyroid-stimulating hormone.

Patients with severe LUTS had a higher incidence of comorbidities, such as hypertension, *diabetes mellitus*, erectile dysfunction, and metabolic syndrome; sedentary conditions; tobacco use; alcohol consumption; perceived stress; and depressive symptoms (all p<0.001), as shown in table 2.

**Table 2:** Relative frequencies of modifiable lifestyle habits and comorbidities in relation to lower urinary tract symptoms

Variable		IPSS			Total n (%)	p value*
		Absent/mild n (%)	Moderate n (%)	Severe n (%)		
Physical activity levels						
	Inactive	3,765 (19.8)	379 (24.9)	58 (29.9)	4,209 (20.3)	<0.001
	Low active	5,569 (29.3)	386 (25.4)	50 (25.8)	6,012 (29.0)	
	Active	7,491 (39.4)	607 (39.9)	70 (36.1)	8,168 (39.4)	
	Very active	2,192 (11.5)	149 (9.8)	16 (8.2)	2,363 (11.4)	
Tobacco use						
	Never	13,426 (70.6)	984 (64.7)	128 (65.8)	14,554 (70.2)	<0.001
	Previous	3,822 (20.1)	408 (26.8)	54 (27.5)	4,271 (20.6)	
	Active	1,769 (9.3)	129 (8.5)	12 (6.7)	1,907 (9.2)	
Alcohol consumption						
	Low-risk	15,917 (83.7)	1,232 (81.0)	157 (80.7)	17,311 (83.5)	0.008
	Hazardous	2,719 (14.3)	242 (15.9)	30 (15.6)	2,985 (14.4)	
	Moderate-severe	380 (2.0)	30 (3.1)	7 (3.6)	435 (2.1)	
Perceiver stress						
	Absent	15,214 (80.0)	1,141 (75.0)	127 (65.6)	16,482 (79.5	<0.001
	Present	3,803 (20.0)	380 (25.0)	67 (34.4)	4,250 (20.5)	
Depressive symptoms						
	Absent	16,545 (87.0)	1,196 (78.6)	126 (65.2)	17,871 (86.2)	<0.001
	Present	2,472 (13.0)	325 (21.4)	68 (34.8)	2,861 (13.8)	
Hypertension						<0.001
	Absent	14,168 (74.50)	951 (62.50)	119 (61.30)	15,238 (73.50)	
	Present	4,849 (25.50)	570 (37.50)	75 (38.70)	5,494 (26.50)	
*Diabetes mellitus*						<0.001
	Absent	17,553 (92.30)	1,311 (86.20)	166 (85.60)	19,032 (91.80)	
	Present	1,464 (7.70)	210 (13.80)	28 (14.40)	1,700 (8.20)	
Erectile dysfunction						<0.001
	Absent	16,545 (87.00)	1,196 (78.60)	126 (65.20)	17,871 (86.20)	
	Present	2,472 (13.00)	325 (21.40)	68 (34.80)	2,861 (13.80)	
Dyslipidemia						0.143
	Absent	8,843 (46.50)	672 (44.20)	83 (42.80)	9,599 (46.30)	
	Present	10,174 (53.50)	849 (55.80)	111 (57.20)	11,133 (53.70)	
Metabolic syndrome						0.021
	Absent	17,039 (89.60)	1,333 (88.00)	165 (85.00)	18,534 (89.40)	
	Present	1,978 (10.40)	182 (12.00)	29 (15.00)	2,198 (10.60)	
Non-alcoholic fatty liver disease						0.136
	Absent	9,204 (48.40)	695 (45.80)	91 (46.90)	9,993 (48.20)	
	Present	9,813 (51.60)	824 (54.20)	103 (53.10)	10,739 (51.80)	

* P values are based on the χ^2^ test.

LUTS: lower urinary tract symptoms; IPSS: international prostate symptom score.

### Factors associated with lower urinary tract symptoms

After constructing a full multiple logistic regression model (Table 1S, Supplementary Material), we performed stepwise backward multiple logistic regression analysis to analyze the variables associated with LUTS. In the final model, depressive symptoms (p<0.001), erectile dysfunction (p<0.001), age (p<0.001) and total PSA levels (p<0.001) were associated with higher odds of LUTS. In contrast, physical activity levels (low active, p<0.001; active, p=0.002; and high active, p=0.005) and total cholesterol levels (p=0.003) were associated with lower odds of developing LUTS, as shown in table 3.

**Table 3:** Predictors of lower urinary tract symptoms

Variable	OR	95% CI		p value*
Physical activity levels (inactive reference)				
Low active	0.71	0.61	0.83	<0.001
Active	0.80	0.69	0.92	0.002
High active	0.74	0.60	0.92	0.005
Depressive symptoms	1.75	1.52	2.01	<0.001
Erectile dysfunction	2.82	2.49	3.18	<0.001
Age	1.06	1.06	1.07	<0.001
TC (mg/dL)	0.99	0.99	0.99	0.003
Total PSA (ng/dL)	1.09	1.07	1.12	<0.001

* P values are based on logistic regression using the backward stepwise selection method.

LUTS: lower urinary tract symptoms; OR: odds ratio; 95%CI: 95% confidence interval; TC: total cholesterol; PSA: prostate-specific antigens.

## DISCUSSION

This study provides a significant evaluation of the regional prevalence and predictive factors of LUTS in Brazil. This is also the only study that has used standardized questionnaires to assess physical activity levels, LUTS, and lifestyle variables. We observed an association between all physical activity levels and LUTS, which differs from the findings of previous studies.^(7–9)^ Our study showed that low-active individuals (OR=0.71, 95%CI=0.61-0.83, p<0.001), active individuals (OR=0.80, 95%CI=0.69-0.92, p=0.002) and high-active individuals (OR=0.74, 95%CI=0.60-0.92, p=0.005) were associated with lower odds of LUTS. Physical activity can decrease low-grade clinical inflammation and increase vascular nitric oxide production, resulting in improved erectile function. Physical activity reduces sexual dysfunction and improves serum testosterone levels.^(7–9)^ Therefore, undertaking regular physical activity, regardless of volume or intensity, can prevent progression of erectile dysfunction and help stabilization or remission of LUTS/benign prostatic hyperplasia. Furthermore, physical activity directly influences other risk factors of LUTS, providing an adjuvant response to the treatment and prevention of this condition.

Our findings also showed that age, depressive symptoms, erectile dysfunction, and total PSA levels were strong independent risk factors for LUTS in men. LUTS is more prevalent among the older population; a high prevalence of LUTS significantly impairs an individual's quality of life and is a predictor of benign prostatic hyperplasia.^(19)^ The pathophysiology of LUTS in older individuals is multifactorial and includes comorbidities and neurological, psychiatric, and behavioral factors.^(20)^ Aging is one of the most important independent factors associated with LUTS, as shown in previous studies^(21,22)^ and as observed in our analysis. Aging affects functional changes in LUTS and other chronic diseases and is associated with reduced bladder capacity, increased uninhibited contractions, decreased urinary flow rate, diminished urethral pressure profile, and increased postvoid residual urine volume.^(23)^

Our study showed that depression was associated with higher odds of developing LUTS than that found in previous studies.^(24,25)^ In a longitudinal study of 9,080 adult Korean men, Rhee et al.^(24)^ reported that LUTS increased the risk of depression by 1.8 times, after adjusting for other factors (p<0.001). A similar association between depression and LUTS was reported in a survey of 5,506 North American men.^(21)^ In this case, depression increased the risk of LUTS by 2.4 times (95%CI= 1.9-3.2, p<0.001). Lower urinary tract symptoms promotes fear, anxiety, worry, confusion, helplessness, and loss of self-confidence, which can result in depression.^(24)^ Furthermore, evidence suggests that the association between LUTS and depression may share common biological pathways such as hormonal levels (serotonin and norepinephrine), systemic inflammation, and the hypothalamic-pituitary-adrenal axis.^(24)^ Additionally, erectile dysfunction is associated with LUTS^(2)^ and depression,^(24)^ as shown in our findings. In our study, erectile dysfunction almost tripled the risk of LUTS. Chronic symptoms of LUTS, including voiding symptoms, play a significant role^(25–27)^ in erectile dysfunction and can be linked to depression. However, the symptoms alone may not explain the longitudinal association with depressive symptoms. Further studies using these outcomes are required.

Another part of this relationship might be explained by the composite mechanism of phosphodiesterase, which induces smooth muscle relaxation by modulating the nitric oxide pathway both at the level of the corpus cavernosum and bladder neck, and the l-Cysteine-H2S pathway at the level of the corpus cavernosum and bladder detrusor, the inhibition of which antagonizes the RhoA/Rho kinase pathway, an important contractive pathway.^(28)^ The patients with LUTS should be evaluated for erectile dysfunction, and the patients presenting with erectile dysfunction be evaluated for LUTS, as this may impact the treatment of the condition.

In addition, our results showed that total PSA levels were associated with higher odds of developing LUTS, which is an important marker of prostate cancer. However, a recent study of 65,000 UK men (50-69 years of age) showed that PSA levels and a history of prostate cancer were not associated with LUTS.^(29)^ The growth of adenoma in benign prostatic hyperplasia appears in the transition zone of the prostate, close to the urethra, and results in LUTS. In contrast, cancer most often develops in the peripheral zone, away from the urethra, indicating a lower likelihood of urinary flow obstruction, especially if the lesions are small.^(30)^ Despite other risk factors for prostate cancer, our results favor a positive association between LUTS and total PSA levels as a clinical marker preventing this pathology.

Finally, our findings regarding the association between total cholesterol and LUTS were conflicting, as higher total cholesterol was associated with lower odds of LUTS (OR=0.99, 95%CI= 0.99-0.99, p=0.003). Some studies have suggested that dyslipidemia is an insufficient predictor of LUTS and benign prostatic hyperplasia.^(21,30)^ Other chronic metabolic changes, such as increased HbA1c, insulin, and type-2 *diabetes mellitus*, may be related to this process.^(30)^ Although statistically significant, the association between total cholesterol and LUTS may have been an incidental finding in this population sample, and further studies are necessary to clarify the role of each type of cholesterol in LUTS.

This study has few limitations. First, causal inferences cannot be made because of the cross-sectional study design. Additionally, only men with private insurance who participated in health checkups were included, which prevents generalization to the entire Brazilian male population and could influence the results of our research. Further, this could have also resulted in selection bias. Although we used standardized questionnaires to measure physical activity and LUTS, the use of questionnaires may also be a potential limitation of our study.

The strengths of this study are that the critical analysis of LUTS and physical activity and standardized health assessments were performed by a physician, including the presence and severity of erectile dysfunction, alcohol consumption, perceived stress, depressive symptoms, and physical activity, which reinforces the reliability and validity of the results. We believe that the results of this study can be used for prevention in both clinical and public health settings. Although our study did not show a causal relationship between physical activity and LUTS, physical activity could influence LUTS-prevention strategies and lifestyle improvements in adults and older men. More studies are warranted to understand the role of physical activity in LUTS in other populations.

## CONCLUSION

We found a strong association between physical activity levels and lower urinary tract symptoms; all categories of physical activity were associated with lower odds of lower urinary tract symptoms. Therefore, physical activity should be strongly encouraged during the treatment and prevention of this condition by public authorities and private clinicians.

## SUPPLEMENTARY MATERIAL Association between physical activity levels and lower urinary tract symptoms: a cohort study in 20,732 Brazilian men

Rafael Mathias Pitta^1^, Oskar Kaufmann^1^, Raphael Mendes Ritti-Dias^2^, Luana de Lima Queiroga^1^, Nelson Wolosker^1,3,4^

**DOI: 10.31744/einstein_journal/2024AO1204**

**Table 1S:** Predictors of lower urinary tract symptoms in men

Variable	OR	95% CI		p value*
Hypertension	1.04	0.93	1.19	0.541
*Diabetes mellitus*	0.91	0.76	1.10	0.336
Metabolic syndrome	0.87	0.72	1.05	0.138
Tobacco				
Previous	0.94	0.82	1.07	0.334
Never	0.87	0.71	1.07	0.198
Physical activity levels				
Low active	0.72	0.61	0.84	<0.001
Active	0.80	0.69	0.93	0.003
High active	0.75	0.61	0.93	0.008
Alcohol consumption				
Hazardous	1.15	0.99	1.35	0.075
Moderate-severe	1.33	0.95	1.86	0.099
Perceived stress	1.09	0.94	1.27	0.263
Depressive symptoms	1.64	1.39	1.92	<0.001
Erectile dysfunction	2.81	2.49	3.17	<0.001
Age	1.07	1.06	1.07	<0.001
BMI (kg/m²)	1.01	0.99	1.03	0.160
TC (mg/dL)	0.99	0.99	0.99	0.04
PSA (ng/dL)	1.10	1.07	1.12	<0.001

* P-values are based on full multiple logistic regression.

OR: odds ratio; 95%CI: 95% confidence interval; BMI: body mass index; TC: total cholesterol; PSA: prostate-specific antigens.
